# Supplementary material for: The interactome of intact mitochondria by cross-linking mass spectrometry provides evidence for coexisting respiratory supercomplexes
Source: Mol Cell Proteomics. 2017 Dec 8;17(2):216–32. doi: 10.1074/mcp.RA117.000470 (PMC5795388; doi:10.1074/mcp.RA117.000470)
Supplement: Supplemental Data [file supp_RA117.000470_133922_0_supp_23978_fzffwf.zip › spectra_annotation/mito_DR_spectra_annotation/249-1-4-1-7-1.pdf]

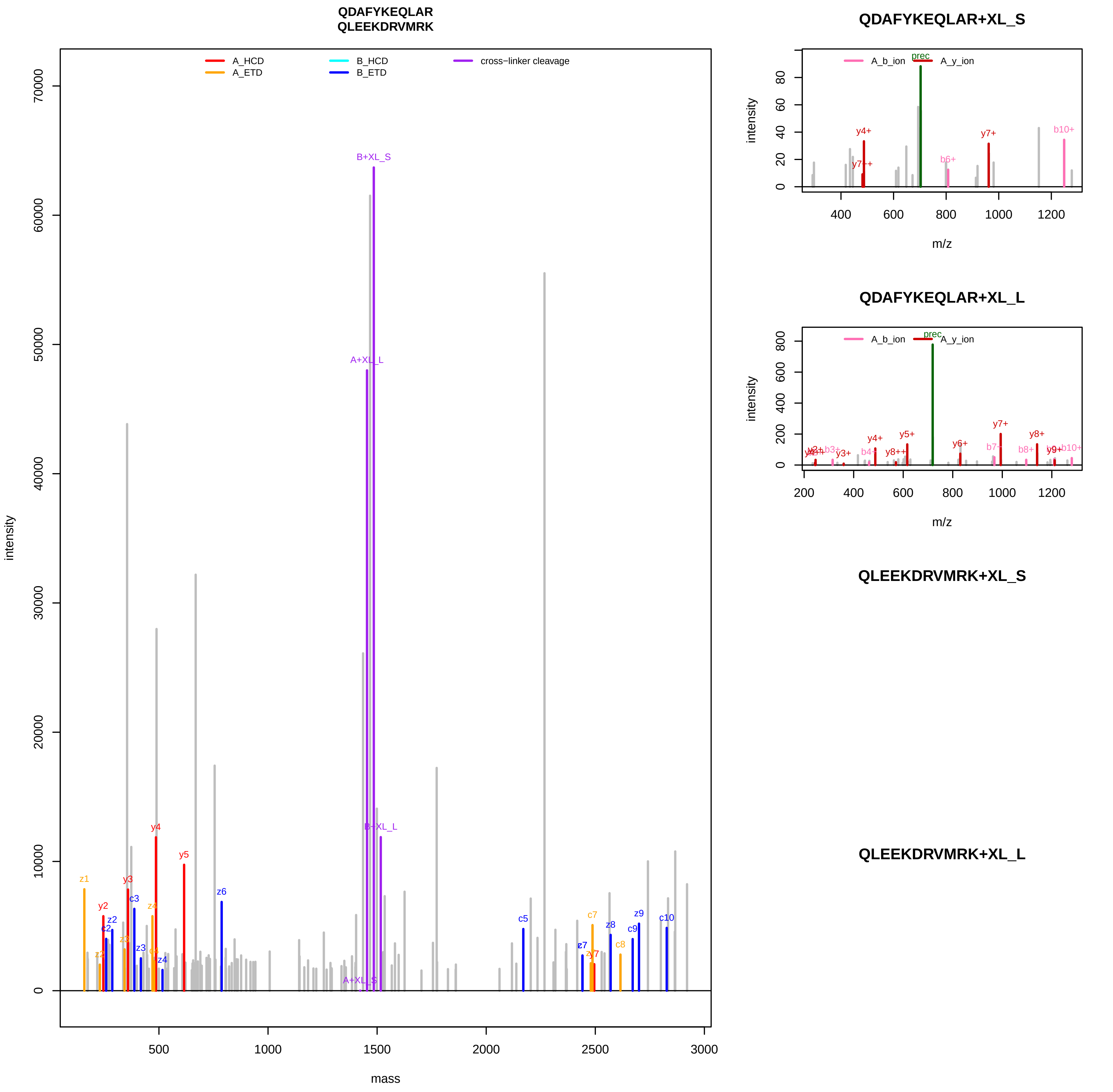
QDAFYKEQLAR
QLEEKDRVMRK
A_HCD
A_ETD
B_HCD
B_ETD
cross−linker cleavage
B+XL_S
A+XL_L
B+XL_L
A+XL_S
y2
y3
y4
y5
z1
z2
z3
z4
c2
c3
c4
z6
c5
c7
c8
z7
z8
z9
c9
c10
intensity
mass
QDAFYKEQLAR+XL_S
A_b_ion
A_y_ion
prec
y4+
y7++
y7+
b6+
b10+
m/z
QDAFYKEQLAR+XL_L
y3+
y4+
y5+
y6+
y7+
y8+
y8++
y9+
b3+
b4+
b7+
b8+
b10+
QLEEKDRVMRK+XL_S
QLEEKDRVMRK+XL_L
